# Study protocol to a nationwide prospective cohort study on return to gainful occupation after stroke in Denmark 1996 - 2006

**DOI:** 10.1186/1471-2458-10-623

**Published:** 2010-10-19

**Authors:** Harald Hannerz, Betina Holbæk Pedersen, Otto M Poulsen, Frank Humle, Lars L Andersen

**Affiliations:** 1 National Research Centre for the Working Environment, 2100 Copenhagen, Denmark; 2 Centre of Rehabilitation of Brain Injury, Amagerfælledvej 56A, 2300 Copenhagen, Denmark

## Abstract

**Background:**

Successful return to work is regarded as one of the most important outcome factors for working-age post stroke patients. The present study will estimate the effect of various predictors on the odds of returning to work after stroke. Nearly twenty thousand 20-57 year-old stroke patients in Denmark who were gainfully occupied prior to the stroke will be included in the study.

**Methods/design:**

Stroke patients will be followed prospectively through national registers. Multi-level logistic regression will be used to model the odds of being gainfully occupied ca. two years after the stroke as a function of the following predictors: Age (20-49 years, 50-57 years) gender, occupational class, self-employment (yes; no), onset calendar year (1996, 1997, ..., 2006), diagnosis (subarachnoid haemorrhage; intracerebral haemorrhage; cerebral infarction; stroke, not specified as haemorrhage or infarction) and 'type of municipality' (the variable is set to 1 if the person lived in a municipality which had a brain injury rehabilitation centre at the time of the stroke. Otherwise it is set to 0).

Municipalities will be treated as the subjects while individual observations within municipalities are treated as correlated repeated measurements.

**Discussion:**

Since our follow-up is done through registers and all people in the target population are included, the study is free from sampling bias, recall bias and non-response bias. The study is also strengthened by its size. The major weakness of the study is that it does not contain any stroke severity measures. Thus, it cannot accurately predict whether a particular stroke patient will in fact return to work. The study is, however, quite useful from a public health perspective. It can be used to estimate the proportion of patients in a certain group that is expected to return to work, and thereby provide a comparison material, which e.g. municipalities can use to evaluate their success in returning their stroke patients to work.

## Background

Successful return to work (RTW) after stroke can enhance both recovery and life satisfaction by consolidating self-esteem, confidence and social identity [1], and it is regarded as one of the most important outcome factors for working-age post stroke patients [2,3].

RTW is of significant importance not only from the human but also from the societal perspective. The consequences of stroke are extremely costly. In 2008, the annual direct cost of stroke in the 27 EU countries was estimated at €18.5 billion, while the indirect cost in terms of lost productivity due to disability or death was estimated at €8.5 billion [4]. In the United States, loss of earnings is expected to be the highest cost contributor in the time period 2005 to 2050 [5].

Recently, Daniel et al. [6] reviewed 70 studies, which reported post-stroke employment status. In total, the 70 studies included 8810 patients working before the stroke. According to Daniel et al., only three of the 70 studies [7-9] used appropriate analytic strategies. "In all other studies, either time of follow-up was highly variable or interpretation of results was compromised by problems with definition of work before stroke and at follow-up or results were likely to be confounded by selection bias" [6]. The three appropriately performed studies were, however, quite small (the sample sizes ranged from 109 to 173) and two of them [7,8] used data from the 1980's, which are too old to afford results that can be used as proxies for present time RTW probabilities. In the most recent of the three studies [9], 55% of previously employed stroke patients returned to paid employment within six months. That study included, however only cognitively competent patients and is therefore selection-biased.

The present study will investigate return to work frequencies among nearly twenty thousand 20 - 57 year-old stroke patients in Denmark who were gainfully occupied prior to the stroke. The study covers stroke that occurred in the time period 1996 - 2006.

## Methods/design

The study will use the Danish Occupational Hospitalisation Register (OHR), a database obtained through a record-linkage between three national registers--the central person register, the hospital register, and the employment classification module. Currently, the OHR includes every person who has been economically active and an inhabitant of Denmark sometime after 1980.

The national hospital register has existed since 1977 and contains data from all public hospitals in Denmark (more than 99% of all admissions). From 1977 to 1994, the register only included inpatients but from 1995 it also covers outpatients and emergency ward visits [10]. The diagnoses have been coded according to international classification of diseases version ten (ICD-10) since 1994.

The central person register contains information on gender, addresses and dates of birth, death and migrations for every person who is or has been an inhabitant of Denmark sometime between 1968 and present time. A person's occupation and social status are, since 1975, registered annually in the employment classification module [10]. The occupations are, since 1994, coded in accordance with Statistics Denmark's Standard Classification of Occupations (DISCO-88) [11], which is a national version of the International Standard Classification of Occupations (ISCO-88). Socio-economic status is coded in accordance with Statistics Denmark's official socio-economic classification [12]. At the one- and two-digit level, the classification contains the following social groups:

1. Gainfully occupied people

1.1. Self-employed people

1.2. Assisting spouses

1.3. Employees

2. People on unemployment benefits

3. Not economically active

3.1. People in training/education

3.2. Pensioners

3.3. Other not economically active

### Inclusion criteria

A person will be included in the study if he/she

1. on at least one occasion in the time period 1996 - 2006, was registered in the hospital patient registry with one of the following ICD-10 codes as principal diagnosis:

• I60 subarachnoid haemorrhage

• I61 intracerebral haemorrhage

• I63 cerebral infarction

• I64 stroke, not specified as haemorrhage or infarction

2. belonged to the age interval 20 - 57 years at the time of the hospital contact

3. was gainfully occupied the year preceding the hospital contact

### Statistical analysis

The study will consist of two parts, one is descriptive while the other utilises statistical inference techniques to test hypotheses and estimate odds ratios for RTW (return to work).

In the descriptive part we follow the stroke patients for five calendar years after the stroke and register their main social status in each of these years. This part of the study will only include people who were less than 55 years at the time of the stroke. For any given patient, the calendar year of the stroke will be defined as year 0, the next calendar year will be defined as year 1 etc.

In the regression analysis, we will look at the odds of having a socio-economic code, which indicates gainful occupation in year 2 after stroke. The outcome variable is set to 1 if the person is self-employed, assisting spouse or employee in that particular year. It is set to 0 if the person is unemployed, not economically active or dead.

As explanatory variables we will use gender, age, diagnosis, calendar year, occupational class, self-employment, and type of municipality.

Age at the time of the stroke will be divided into the categories 20 - 49 years and 50 - 57 years. In Denmark it is possible to retire at the age of 60, regardless of health condition. This is why we do not include people who would be older than 60 years at follow-up. The cut-point 50 years conforms to OECD's definition of older workers [13], who are known to have a more insecure labour market attachment than the younger ones.

The variable 'Diagnosis' contains the four stroke categories given in the section 'inclusion criteria'.

The variable 'Self-employment' is set to 1 if the person is self-employed or assisting spouse and 0 if he/she is an employee, the year preceding the stroke.

The variable 'Occupational class' is based on the first digit of the DISCO-88 classification the year preceding the stroke. It contains the following categories:

• Legislators, senior officials and managers (DISCO-88 group 1)

• professionals (DISCO-88 group 2)

• technicians and associate professionals (DISCO-88 group 3)

• workers in occupations that require skills at a basic level (DISCO-88 group 4 - 8)

• workers in elementary occupations (DISCO-88 group 9)

• gainfully occupied people with an unknown occupation (missing DISCO-88 code)

The variable 'Type of municipality' is set to 1 if the person lived in a municipality which had a brain injury rehabilitation centre at the time of the stroke. Otherwise it is set to 0. The following municipalities had a brain injury rehabilitation centre throughout the study period: Copenhagen, Odense, Aarhus, Roskilde, Aalborg and Vejle.

There are at least two reasons for believing that the RTW probabilities depended on which calendar year the stroke occurred. Firstly, quality of stroke treatment and rehabilitation has a tendency to improve with time. This is illustrated by two Finish studies which show that 28-day case fatality rates among 35 - 74 year-old stroke patients decreased by approximately three percent annually in the time period 1983 - 2001 [14,15]. A similar trend was observed in England [16]. Secondly, a series of political initiatives and legislative changes, aimed at improving return to work rates in Denmark, occurred during the study period. In 1998, the flexi-job system was introduced, which allows people with permanently reduced work capacity to work part time, yet through public subsidy get full salary. In 2001, the flexi-job system was reformed. A person who is eligible to a flexi-job would be entitled to unemployment benefits if no such work could be found. In 2003, a new disability retirement scheme was introduced. The main intent of the reform was to ascertain that as many as possible retain their attachment to the labour market. A new procedure for assessing work capacity was introduced, and disability pension would only be granted if work ability was permanently reduced and flexi-job work was unfeasible. In 2005, the municipal control of the sick-listed was intensified by a change in the Sickness Benefit Act. Follow-up evaluations were required once a month instead of once every second month, and a reintegration plan was to be drawn up after four instead of six months [17]. To deal with this possible time dependency, we incorporate calendar year into the model as a class variable.

It is also reasonable to believe that RTW probabilities depend on place of residence. In Denmark, municipalities play an important role in the return to work process. According to the law, it is the municipal officer and not the physician who has the formal right to decide whether or not a person qualifies for sickness benefit, disability pension, or vocational rehabilitation. The law also stipulates that the municipality should perform regular follow-up evaluations and draw up detailed reintegration plans for each sick-listed citizen at risk of long-tem sickness absence [17]. RTW initiatives are often launched at the municipality level and some municipalities might be more active than others. The unemployment situations may also differ between municipalities. In the present study, we will use a multi-level analysis to deal with intra-municipality correlations -- the municipalities are treated as the subjects while the individual observations within the municipalities are treated as correlated repeated measurements.

The logistic regression will be performed by use of the GENMOD procedure in SAS version 9.1. Only main effects are considered. An exchangeable correlation structure is assumed. The empiric standard error estimates will be used.

Table 1 shows how the results from the first part of the study will be presented. Table 2 will list odds ratios and P-values that will be estimated in the second part of the study. Odds ratio for return to gainful occupation two year after stroke, by onset calendar year, will be illustrated by a graph. The calendar year 1996 will be used as reference. All hypotheses are two-tailed. A P-value will be deemed statistically significant if it is less than 0.05.

**Table 1:** Social group distribution (%) by time passed since onset of illness, among stroke patients in Denmark who were 20-54 year of age and gainfully occupied at the time of the stroke

Social status	Year after stroke				
	1	2	3	4	5
Self-employed people					
Assisting spouses					
Employees					
People on unemployment benefits					
People in training/education					
Pensioners					
Other not economically active					
Deceased					

Total	100	100	100	100	100

**Table 2:** Odds ratios (OR), with 95% confidence interval (CI) for return to gainful occupation two year after stroke

Parameter	Level	N	OR	95% CI
Gender (P = xxx)	Men		1.00	-
	Women			
Age (P = xxx)	<50 years		1.00	-
	50 - 57 years			
Diagnosis (P = xxx)	Subarachnoid haemorrhage			
	Intracerebral haemorrhage			
	Cerebral infarction		1.00	-
	Stroke, not specified as haemorrhage or infarction			
Self-employment (P = xxx)	No		1.00	-
	Yes			
Occupational class (P = xxx)	Legislators, senior officials and managers			
	Professionals			
	Technicians and associate professionals			
	Workers in occupations that require skills at a basic level			
	Workers in elementary occupations		1.00	-
	Gainfully occupied people NOS			
Municipality type (P = xxx)	Municipality without brain injury centre		1.00	-
	Municipality with brain injury centre			

### Power calculations

We include all stroke patients of the concerned population and time period. The sample size is therefore fixed at ca. 20 000 persons. The statistical power of the study depends on which factor we consider and what effect sizes we wish to detect. The factor associated with the least power would be self-employment (only ten percent of the concerned stroke patients are self-employed). If we assume that 55% of the stroke patients have returned to stable employment two years after stroke and that intra-municipality correlations inflate the variance with 20% then (based on the central limit theorem and the propagation of error formulas) we have at least a 95% chance of detecting an effect of self-employment if the true odds-ratio between the self-employed and other employees is either greater than 1.3 or less than 1/1.3.

## Discussion

Our follow-up is done prospectively through registers and does not require that the participants should fill in a questionnaire. Hence we do not have any problems with recall bias or non-response bias. Since informed consent from the participants is not required for register studies of the present type, the study is free from volunteer bias. It is also free from sampling bias since all people in the target population are included. Another advantage of the study is that referral bias is minimal; the diagnoses under study are of a kind that requires hospital treatment. The study is further strengthened by its size.

A drawback of the Occupational Hospitalisation Register is that the occupational and socio-economic data are given per calendar year rather than on a daily basis. We know the person's main occupation and social group during a calendar year but we cannot know the exact date when he returns to work. Due to this shortcoming, a person who returns to work in a given calendar year would still be treated as a non-returner in the analysis if he, for example, was unemployed during the major part of that year. The participants are in other words required to return to stable gainful employment before they are categorised as having returned to work. This can, however, also be regarded as a strength; it has been shown that many people with brain injury who return to work are unable to continue their employment [1].

Overall stroke severity is the most consistent predictive factor for RTW [18]. The major weakness of the present study is that it does not include any such measure. Data on post-stroke neurological impairments such as the presence and degree of apraxia, aphasia and agnosia, would definitely improve our chances of correctly guessing whether or not an individual patient would return to work. Our study is, however, quite useful from a public health perspective. It can be used to estimate the proportion of patients in a certain group that is expected to return to work and thereby provide a comparison material, which e.g. municipalities can use to gauge their success in returning their stroke patients to work.

Another limitation of the study concerns the variable 'municipality type'. The study can tell us whether or not stroke patients in municipalities with brain injury rehabilitation centres have a better prognosis than those in other municipalities but it cannot tell us if this is due to the centres. That question can only be answered through a randomised study.

## Competing interests

Frank Humle, one of the authors, is Director of the Centre for Rehabilitation of Brain Injury (CRBI) in Copenhagen, Denmark. CRBI is a self-owned fund which is financially supported by grants from the Danish municipalities and, to a lesser degree, by a collectively bargained framework agreement under the Danish Health Law that covers 20% of the funds' operation costs. Since the study can tell us whether or not stroke patients in municipalities with brain injury rehabilitation centres have a better prognosis than those in other municipalities, there is a potential conflict of interest. We believe, however, that any potential bias due to competing interests is eliminated with the publication of this protocol, which implies a commitment to adhere to the methods chosen and to publish the results regardless of the outcome.

## Ethics approval

The study will comply with The Act on Processing of Personal Data (Act No. 429 of 31 May 2000), which implements the European Union Directive 95/46/EC on the protection of individuals. The data usage is approved by the Danish Data Protection Agency, journal number: 2001-54-0180. According to Danish law, questionnaire and register based studies do not need approval by ethical and scientific committees, nor informed consent.

## Authors' contributions

LLA, OMP and FH initiated the project and acquired the funding. LLA, OMP and HH designed the study. HH prepared the first draft of the manuscript. All authors contributed in a critical revision of the manuscript. All authors have given their final approval of the version submitted for publication.

## Pre-publication history

The pre-publication history for this paper can be accessed here:

http://www.biomedcentral.com/1471-2458/10/623/prepub
